# Effects of Nutrition and Exercise Interventions on Persons with Sarcopenic Obesity: An Umbrella Review of Meta-Analyses of Randomised Controlled Trials

**DOI:** 10.1007/s13679-023-00509-0

**Published:** 2023-05-30

**Authors:** Lea Reiter, Silvia Bauer, Mariella Traxler, Josje D. Schoufour, Peter J. M. Weijs, Alfonso Cruz-Jentoft, Eva Topinková, Doris Eglseer

**Affiliations:** 1 Medical University of Graz, Institute of Nursing Science, Neue Stiftingtalstraße 6 West, P/06, 8010 Graz, Austria; 2 Faculty of Sports and Nutrition, Centre of Expertise Urban Vitality, Amsterdam University of Applied Sciences, Dr. Meurerhuis, Dokter Meurerlaan 8, 1067 SM Amsterdam, Netherlands; 3 Department of Nutrition and Dietetics, Amsterdam University Medical Centers, Amsterdam Public Health Institute, VU University, Amsterdam, Netherlands; 4 Servicio de Geriatría, Hospital Universitario Ramón Y Cajal (IRYCIS), Ctra. Colmenar Viejo, 28034 Madrid, Spain; 5 Charles University Department of Geriatric Medicine, First Faculty of Medicine, Londynska 15, Praha 2, 120 00 Prague, Czech Republic

**Keywords:** Dietary protein, Resistance training, Review, GRADE approach, Sarcopenia, Obesity

## Abstract

**Background:**

Sarcopenic obesity (SO) is an increasing phenomenon and has been linked to several negative health consequences. The aim of this umbrella review is the assessment of effectiveness and certainty of evidence of nutrition and exercise interventions in persons with SO.

**Method:**

We searched for meta-analyses of RCTs in PubMed, EMBASE and CENTRAL that had been conducted in the last five years, focusing on studies on the treatment and prevention of SO. The primary endpoints were parameters for SO, such as body fat in %, skeletal muscle mass index (SMMI), gait speed, leg strength and grip strength. The methodological quality was evaluated using AMSTAR and the certainty of evidence was assessed using GRADE.

**Results:**

Four systematic reviews with between 30 to 225 participants were included in the umbrella review. These examined four exercise interventions, two nutrition interventions and four interventions that combined nutrition and exercise. Resistance training was the most frequently studied intervention and was found to improve gait speed by 0.14 m/s to 0.17 m/s and lower leg strength by 9.97 kg. Resistance, aerobic, mixed exercise and hypocaloric diet combined with protein supplementation is not significantly effective on selected outcomes for persons with SO compared to no intervention. The low number of primary studies included in the reviews resulted in moderate to very low certainty of evidence.

**Conclusion:**

Despite the lack in certainty of evidence, resistance training may be a suitable intervention for persons with SO, in particular for improving muscle function. Nevertheless, further research is necessary to strengthen the evidence.

**Supplementary Information:**

The online version contains supplementary material available at 10.1007/s13679-023-00509-0.

## Introduction

Ageing is associated with a variety of changes in body composition, such as an increase in fat accumulation as well as a loss of muscle mass and strength [1–3]. Consequently, with increasing age there is an increased risk of obesity, which is the excessive accumulation of fat [4], as well as the risk of sarcopenia, which is characterised by low muscle mass and muscle function [5]. Both sarcopenia and obesity can occur simultaneously and synergistically aggravate each other, resulting in sarcopenic obesity [5]. The health consequences of sarcopenic obesity can be worse than for sarcopenia or obesity alone. Researchers have shown that sarcopenic obese individuals are 2.5 times more at risk of disability than individuals with sarcopenia or obesity alone [6]. Furthermore, sarcopenic obesity has been linked to several negative health consequences, such as an increased risk of falling, cardiovascular diseases, comorbidity and early mortality, as well as institutionalisation [7].

The European Society for Clinical Nutrition and Metabolism (ESPEN) and the European Association for the Study of Obesity (EASO) recently reached a consensus on the definition of sarcopenic obesity, stating it being the co-existence of obesity and sarcopenia. More specifically, the defining criteria are an increased body mass index or waist circumference as well as the simultaneous occurrence of increased fat mass, low muscle mass and low muscle strength and function. However, it is not yet clear if combining the separate definitions of obesity and sarcopenia is applicable for persons with sarcopenic obesity [8••]. Nonetheless, a recently published systematic review and meta-analysis showed that the pooled global prevalence of sarcopenic obesity is at 11% [9•]. Furthermore, it must be taken into consideration, that sarcopenic obesity often is overlooked due to the fact that high fat mass masks low muscle mass [10•].

As well as reducing the accompanied complications such as cardiovascular disease, the main goals when treating sarcopenic obesity are to decrease body fat and to support the build-up or preservation of muscle mass and muscle function [5, 11••, 12]. Several clinical trials have shown that nutrition interventions and physical exercise improve relevant parameters regarding sarcopenic obesity such as grip strength, body fat and muscle mass [5, 13–17]. Furthermore, whole-body electromyostimulation (WB-EMS) has emerged in research as a useful treatment for sarcopenia [18] and sarcopenic obesity, showing promising effects due to its similarities to resistance training [5, 19, 20]. Currently, few systematic reviews are available on the efficacy of nutrition and exercise interventions as treatment strategies for sarcopenic obesity. The available reviews mainly address the efficacy of exercise interventions and/or nutrition interventions as a whole, instead of studying individual interventions such as resistance training or increased protein intake [21, 22]. Therefore, the question of an optimal treatment strategy for sarcopenic obesity is left out and remains unanswered. To our knowledge, the current study represents the first umbrella review on sarcopenic obesity conducted to provide comprehensive insights into the effectiveness of different nutrition and exercise interventions for adults, therefore contributing essential findings that will enable future researchers to devise an optimal treatment strategy for individuals with sarcopenic obesity.

## Methods

To address the research question, we conducted an umbrella review in accordance with the Preferred Reporting Items for Systematic Reviews and Meta-analyses (PRISMA) guidelines [23]. This umbrella review was registered at PROSPERO (CRD42022342822).

## Search Strategy

We conducted a comprehensive literature search to identify systematic reviews and meta-analyses available in PubMed via Medline, EMBASE, and the Cochrane Library (Cochrane Database of Systematic Reviews) via OVID.

We used the following search terms to identify relevant meta-analyses: “obese sarcopen*”, “sarcobesity”, “sarcopenic obes*”, “train*”, “physical activity”, “exercise”, “diet”, “nutr*” and “energy restriction”. We also applied the MeSH terms “obesity”, “sarcopenia”, “exercise”, “Diet, Food, and Nutrition” and “Nutrition Therapy”. The search terms were combined using the Boolean operators AND or OR. Where possible, filters were set regarding the publication type (“systematic review” and “meta-analyses”) and age (> 18 years). We also narrowed the search down to the last five years. This limitation was set as sarcopenic obesity is a relatively new topic in research. Furthermore, the age limit was set to 45 and older in order to find reviews specifically focusing on adults. No language restriction was applied. The exact search strategy can be found in the Online Resource 1. Furthermore, we conducted a manual search in Google Scholar by using the search terms “sarcopenic obesity systematic review” and screened the first five pages of results. In addition, we screened the reference lists of relevant studies to find further studies. In August 2022, we conducted an update search to find additional reviews which had been published since May 2022. In doing so, we applied the exact same search strategy in the same databases and applied the relevant time filter where possible. No additional systematic reviews were identified as a result of the update search.

## Eligibility Criteria

For a systematic review to be included in this umbrella review, the following criteria had to be met: meta-analysis of RCTs and a comparison of any exercise or nutrition intervention with a control group of adults with diagnosed sarcopenic obesity. The outcomes of interest were based on the definition of the terms *sarcopenia* and *obesity*; therefore, the following parameters were selected: body fat in %, total body fat mass, total muscle mass in kg, appendicular skeletal muscle mass (ASMM) in kg, skeletal muscle mass index (SMMI), lower/leg extremity strength, grip strength and gait speed.

Two independent reviewers (M.T., S.B.) selected titles, abstract and full texts according to the inclusion criteria. If disagreements arose, a consensus was reached by discussion, and, if necessary, a third person was consulted to make a final decision.

## Data Extraction and Synthesis

Data were extracted from the final full texts which remained after screening was performed. The extraction process was conducted by two authors (M.T., S.B.) using a data extraction sheet. In addition to extracting the general characteristics (Table 1) of the research from the studies, we extracted the following data for each outcome: type of intervention, number of studies which were pooled per outcome, sample size according to intervention group (IG) and control group (CG), metric (standardized mean difference or mean difference), effect-size (CI 95%), *I*_2_ and *p*-value. If data were not stratified according to type of intervention (i.e. resistance training vs control; aerobic training + protein supplementation vs control), we analysed the table of study characteristics to group primary studies which measured the same types of interventions and outcomes. Subsequently, data from the primary studies with the same type of intervention were then pooled in RevMan 5.4., and the effect sizes were calculated as mean differences or standard mean differences, depending on the outcome. The confidence interval was set to 95%, and a *p*-value < 0.05 was considered as statistically significant. Furthermore, we applied the random-effects model and assessed the heterogeneity (*I*^2^).

**Table 1:** Characteristics of the included reviews with meta-analyses

**Author (Year)**	**No. of included primary studies**^**a**^	Total sample size*	**Age range (years)**	**Sex**	**Diagnostic criteria of sarcopenia/obesity**	**Type of intervention**	**Duration (weeks)**
Eglseer et al. [27••]	12	574	58 to 74.1	9 studies included only women 2 included men and women 1 included only men	**Sarcopenia**: SMI (ASM/BW, TSM/BW, ASM/Ht2, TSM/Ht2), HGS **Obesity**: BMI, VFA or BF %	**Exercise alone** (aerobic, resistance, combined) or **Nutrition alone** (caloric restriction, high protein, isoflavones, olive oil combined with healthy Brazilian diet) **Exercise plus nutrition** (resistance training and protein supplementation)	8–52
Hita-Contreras et al. [21]	9 (7 included in meta-analyses)	692	66.8 to 81.4	5 studies included only women 3 included only men 1 included men and women	**Sarcopenia**: low SMM by ASM, AFFM relatives to Ht_2_, weight/BMI **Obesity**: BMI or % in body fat or VFA	**Exercise** (aerobic, resistance, combined, WBEMS) or **Exercise plus nutrition** (protein supplementation – high leucine, vitamin D, tea catechin) **Mixed training** (WBEMS and aerobic, resistance und aerobic)	8–26
Hsu et al. [26]	15	748	41 to 90	10 studies included only women 2 included only men 3 included men and women	**Sarcopenia**: SMI (ASM/Ht_2_, TSM/Ht_2_, ASM/BW, TSM/BW, ASM/BMI, FFM) **Obesity**: BMI or BF%	**Exercise alone** (aerobic, resistance, combined exercise, power training) or **Nutrition alone** (supplementation, LCHP, low-calorie-normal protein diet, isoflavone, protein supplementation, amino acid supplementation, catechin-fortified tea **Exercise plus nutrition** (supplementation and exercise, dairy/non-dairy isocaloric and isoprotein supplementation and resistance exercise, supplementation and combined exercise, 4-month protein supplementation and resistance exercise)	8–24
Yin et al. [22]	12 (10 included in meta-analyses)	863	41 to 90	8 studies included only women 2 included men and women 2 included only men	**Sarcopenia:** HGS, gait speed, low muscle mass, AFFM, FMM, appendicular lean soft tissue **Obesity:** BMI, body fat in %, VFA, WC, FMI	**Exercise alone** (aerobic, resistance, elastic resistance, combined, WB-EMS, power circuit training) **Nutrition alone** (protein supplementation, low calorie + high protein intake) **Exercise plus nutrition** (combined exercise + supplementation, WB-EMS + supplementation, aerobic training + protein, resistance training + protein)	8–28

*BMI*  Body Mass Index, *SMI*  skeletal muscle index, *SMM*  skeletal muscle mass, *ASM*  appendicular skeletal mass, *AFFM*  appendicular fat free mass, *BW*  body weight, *Ht*_*2*_squared body height, *TSM*  total skeletal mass, *FFM*  fat free mass, *VFA*  visceral body fat, *LCHP*  low-calorie high-protein, *HGS*  handgrip strength, *WB-EMS*  Whole-Body electromyostimulation, *FM*  fat mass, *FMI*  fat mass index, *LLM*  leg lean mass, *LP*  leg press, *MT*  muscle thickness

*sample sizes of meta-analyses are based on included outcome

^a^primary studies included in the reviews are partially overlapping

Newly calculated effect sizes are noted accordingly in the data extraction table. Studies which assessed the effects of the same intervention and outcome were grouped together in this table.

## Assessment of Methodological Quality of the Systematic Reviews

We used the Assessment tool AMSTAR 2 (A Measurement Tool to Assess Systematic Reviews) to assess the methodological quality of the systematic reviews. This tool is comprised of 16 items of which seven (7) items (2, 4, 7, 9, 11, 13, 15) are considered as critical, meaning they have influential impact on the review’s quality. The overall rating of the AMSTAR can be categorised as high, moderate, low, or critically low and is based on the rating of critical items. [24] The appraisal was conducted by two authors (M.T., S.B.) independently, and discrepancies were resolved until a consensus was met. The rating assigned to each study is shown in Table 2*.*

**Table 2:** Results of AMSTAR assessment

Authors	No. of 1	No. of 2*	No. of 3	No. of 4*	No. of 5	No. of 6	No. of 7*	No. of 8	No. of 9*	No. of 10	No. of 11*	No. of 12	No. of 13*	No. of 14	No. of 15*	No. of 16	AMSTAR 2
Eglseer et al. [27••]	Y	PY	N	PY	Y	Y	PY	Y	Y	N	Y	Y	Y	Y	Y	Y	Moderate
Hita-Contreras et al. [21]	Y	N	N	PY	Y	Y	PY	Y	Y	N	Y	N	N	N	Y	Y	Low
Hsu et al. [26]	Y	N	N	PY	N	Y	PY	Y	Y	N	Y	N	N	N	N	Y	Critically low
Yin et al. [22]	Y	Y	N	PY	Y	Y	PY	Y	Y	N	Y	N	Y	Y	N	Y	Low

Y = Yes, PY = Partial yes, N = No, * = Critical item

## Assessment of the Quality of the Evidence

To assess the quality of the evidence of the included studies, we used the Grading of Recommendation Assessment, Development and Evaluation (GRADE), which classifies the strength of evidence as high, moderate, low, or very low. These included the following points: (1) risk of bias in the individual studies, (2) inconsistency, (3) indirectness, (4) imprecision and (5) publication bias. [25] Reviews which included only one primary study for an intervention were automatically rated as very imprecise, as heterogeneity (*I*^2^) was not applicable. Instead of assessing the risk of bias per outcome, the risk of bias was rated for an entire review. Two reviewers (D.E., S.B.) who had had experience working with GRADE assessed the quality of the evidence for each outcome. Disagreements were discussed until a consensus was met, and, if necessary, a third person was consulted (L.R.) to arrive at a final decision.

## Results

**Fig. 1:**
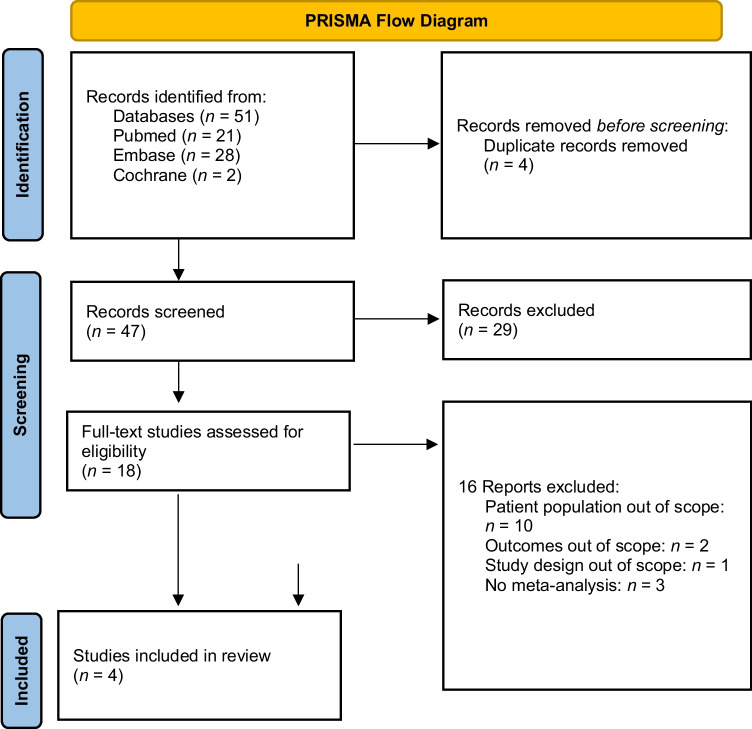
PRISMA flowchart showing screening process applied during the systematic literature search

As shown in Fig. 1, the search enabled us to identify 51 systematic reviews and meta-analyses. After removing duplicates (*n* = 4), 47 articles were included in the title and abstract screening. Following, 18 full texts were assessed for their eligibility, resulting in the inclusion of two reviews for data extraction [21, 26]. A manual search resulted in the identification of two further studies which were deemed eligible for our review [22, 27••]. The reasons for excluding studies can be found in the *PRISMA flow chart* (Fig. 1)*.*

All four of the included studies were systematic reviews focussing on people with diagnosed sarcopenic obesity [21, 22, 26, 27••].

A total of ten interventions were identified in these reviews, representing either exercise or nutrition interventions or a combination of these. Exercise interventions included resistance training, aerobic training, WB-EMS (whole-body electromyostimulation) and mixed training (resistance training and aerobic training). Nutrition interventions comprised mainly of protein supplementation; and low-calorie diets combined with high-protein intake diets. The combinations of nutrition and exercise interventions were WB-EMS and protein supplementation, mixed training and protein supplementation, resistance training and protein supplementation. No review was found that studied caloric restriction in combination with exercise and/ or protein supplementation. The study characteristics can be seen in Table 1.

## Methodological Quality

According to the AMSTAR 2 assessment results regarding the quality of the reviews, only one was rated as being of moderate quality [27••], two systematic reviews were of low quality [21, 22] and one was rated as being critically low [26]. The methodological quality of the included studies is shown in Table 2. While three of the seven critical items were rated with “yes” (criterion met) or “partial yes” for all studies, the critical items no. 2 (methodology in protocol), 13 (discussing risk of bias) and 15 (publication bias) apparently contributed to the flaws in quality. Both Hita-Contreras et al. [21] and Hsu et al. [26] had not preliminarily defined the methodology used for their reviews in a protocol (item no. 2) and also failed to take the risk of bias into consideration when interpreting the results, (item no. 13) resulting in items no. 2 and no. 13 being rated with “no” (criterion not met). Regarding a possible publication bias (item no. 15), neither Hsu et al. [26] nor Yin et al. [22] performed any statistical or graphical analyses to detect a publication bias. Furthermore, Hsu et al. [26] was the only review that did not perform the study selection through two reviewers independently, which additionally contributed to the critically low quality of the review. Eglseer et al. [27••] which was the only review with moderate quality, was also the only review to take the risk of bias into consideration when interpreting results of the meta-analyses.

## Exercise Interventions

### Effect of Resistance Training

All reviews that measured gait speed and lower/leg extremity strength reported that significant improvements were observed with resistance training. The effect of resistance training on gait speed was studied in three reviews, with two reviews revealing significant improvements ranging from 0.14 (95% CI, 0.05; 0.23) to 0.17 (95% CI, 0.01; 0.34) (CoE low) [22, 26, 27••]. Lower/leg extremity strength was analysed only by Eglseer et al. [27••] and showed a significant improvement of 9.97 kg (95% CI, 4.43; 15.51) (CoE low) (see Table 3).

**Table 3:** Extracted data from reviews studying interventions regarding exercise, nutrition and a combination of exercise and nutrition

**Author**	**Intervention**	**Included Primary Studies**^**c**^	**Sample Size per Outcome**		**Effect (95% of CI)**	***p*-value**	***I***^***2***^**%**	**GRADE Confidence in Evidence**	**AMSTAR Rating**
**Resistance Training**									
Eglseer et al. [27••]	Body fat in %	4	IG 104	CG 94	-1.53 (-2.91 to -0.15)	0.03	28%	Moderate	Moderate
Hita-Contreras et al. [21]		3	IG 102	CG 96	-0.56 (-1.46 to 0.34)^b^	0.22	0%	Low	Low
Hsu et al. [26]		5	IG 121	CG 104	-2.67 (-4.03 to -1.32)	< 0.05	17%	Very low	Critically low
Yin et al. [22]		3	IG 117	CG 102	-1.82 (-4.29 to 0.65)	0.15	66%	Moderate	Low
Hita-Contreras et al. [21]	Total body fat mass in kg	2	IG 33	CG 32	-1.45 (-5.04 to 2.14)^b^	0.43	0%	Low	Low
Hsu et al. [26]		3	IG 58	CG 53	-3.28 (-5.63 to-0.94)	0.006	0%	Very low	Critically low
Eglseer et al. [27••]	Total muscle mass in kg	3	IG 73	CG 63	-0.01 (-0.98 to 0.96)	0.99	11%	Very low	Moderate
Hsu et al. [26]		4	IG 88	CG 73	0.36 (-0.96 to 1.68)	0.59	0%	Very low	Critically low
Hita-Contreras et al. [21]	SMMI	1	IG 15	CG 15	0.18 (-0.54 to 0.89)^b^	0.63	n.a.*	Low	Low
Yin et al. [22]		2	IG 48	CG 38	0.28 (-0.15 to 0.71)	0.20	0%	Moderate	Low
Eglseer et al. [27••]	Grip strength in kg	3	IG 73	CG 63	3.73 (2.60 to 4.85)	< 0.0001	0%	Moderate	Moderate
Hita-Contreras et al. [21]		1	IG 15	CG 15	6.00 (-1.68 to 13.68)^b^	0.13	n.a.*	Low	Low
Hsu et al. [26]		3	IG 73	CG 67	4.52 (1.88 to 7.17)	0.0008	0%	Very low	Critically low
Yin et al. [22]		2	IG 48	CG 38	2.88 (-0.88 to 6.64)	0.13	46%	Moderate	Low
Eglseer et al. [27••]	Lower/leg extremity strength in kg	1	IG 14	CG 14	9.97 (4.43 to 15.51)	0.0004	n.a*	Low	Moderate
Eglseer et al. [27••]	Gait speed in m/s	3	IG 72	CG 62	0.17 (0.01 to 0.34)	0.04	85%	Low	Moderate
Hsu et al. [26]		3	IG 69	CG 55	0.23 (0.00 to 0.46)	0.05	91%	Very low	Critically low
Yin et al. [22]		1	IG 33	CG 23	0.14 (0.05 to 0.23)	0.002	n.a.*	Low	Low
**Aerobic Training**									
Hita -Contreras et al. [21]	Body fat in %	1	**IG** 15	**CG** 15	-1.70 (-5.90 to 2.50^b^	0.43	n.a*	Low	Low
Hsu et al. [26]		1	**IG** 15	**CG** 15	-1.50 (-4.83 to 1.83)	0.38	n.a*	Very low	Critically low
Yin et al. [22]		1	**IG** 15	**CG** 15	-1.70 (-4.99 to 1.59)	0.31	n.a*	Low	Low
Hita -Contreras et al. [21]	Total body fat mass in kg	1	**IG** 15	**CG** 15	-1.50 (-6.99 to 3.99)^b^	0.59	n.a.*	Low	Low
Hsu et al. [26]		1	**IG** 15	**CG** 15	-5.20 (-9.47 to -0.93)	0.02	n.a.*	Very low	Critically low
Hsu et al. [26]	Total muscle mass in kg	1	**IG** 15	**CG** 15	-1.00 (-3.51 to 1.51)	0.44	n.a.*	Very low	Critically low
Hita -Contreras et al. [21]	SMMI	1	**IG** 15	**CG** 15	0.26 (-0.46 to 0.98)^b^	0.48	n.a.*	Low	Low
Yin et al. [22]		1	**IG** 15	**CG** 15	0.33 (-0.39 to 1.05)	0.37	n.a.*	Low	Low
Hita -Contreras et al. [21]	Grip strength in kg	1	**IG** 15	**CG** 15	-0.40 (-8.08 to 7.28)^b^	0.92	n.a.*	Low	Low
Hsu et al. [26]		1	**IG** 15	**CG** 15	-0.50 (-6.22 to 5.22)	0.86	n.a.*	Very low	Critically low
Yin et al. [22]		1	**IG** 15	**CG** 15	-0.40 (-6.35 to 5.55)	0.90	n.a.*	Low	Low
**Mixed Training**									
Hita -Contreras et al. [21]	Body fat in %	3	**IG** 75	**CG** 74	-1.63 (-3.53 to 0.27)^b^	0.09	0%	Low	Low
Hsu et al. [26]		3	**IG** 74	**CG** 74	-2.05 (-3.50 to -0.61)	0.005	0%	Very low	Critically low
Yin et al. [22]		3	**IG** 74	**CG** 74	-1.63 (-3.30 to 0.03)	0.05	23%	Moderate	Low
Hita -Contreras et al.[21]	Total Body Fat Mass in kg	2	**IG** 50	**CG** 49	-0.40 (-3.08 to 2.27)^b^	0.77	0%	Low	Low
Hsu et al. [26]		2	**IG** 49	**CG** 49	-2.34 (-4.26 to -0.43)	0.02	66%	Very low	Critically low
Hsu et al. [26]	Total muscle mass in kg	1	**IG** 15	**CG** 15	0.20 (-2.48 to 2.88)	0.88	n.a.*	Very low	Critically low
Hita -Contreras et al. [21]	ASMM in kg	2	**IG** 60	**CG** 59	0.22 (-0.69 to 1.13)	0.63	0%	Low	Low
Yin et al.[22]		2	**IG** 59	**CG** 59	0.25 (-0.47 to 0.98)	0.50	0%	Moderate	Low
Hita -Contreras et al. [21]	SMMI	2	**IG** 50	**CG** 49	-0.03 (-0.56 to 0.51)^b^	0.96	38%	Low	Low
Yin et al. [22]		2	**IG** 49	**CG** 49	0.02 (-0.61 to 0.66)	0.94	55%	Moderate	Low
Hita -Contreras et al. [21]	Grip strength	3	**IG** 75	**CG** 74	1.86 (-1.04 to 4.75)^b^	0.21	59%	Moderate	Low
Hsu et al. [26]		3	**IG** 74	**CG** 74	2.33 (-1.63 to 6.30)	0.25	88%	Very low	Critically low
Yin et al. [22]		3	**IG** 74	**CG** 74	1.71 (-1.25 to 4.68)	0.26	78%	Low	Low
Hita -Contreras et al. [21]	Gait speed in m/s	2	**IG** 60	**CG** 59	0.12 (0.02 to 0.22)^b^	0.02	0%	Moderate	Low
Hsu et al. [26]		2	**IG** 59	**CG** 59	0.15 (0.04 to 0.26)	0.006	51%	Very low	Critically low
Yin et al. [22]		2	**IG** 59	**CG** 59	0.14 (0.05 to 0.23)	0.002	27%	Moderate	Low
**WB-EMS**									
Hita -Contreras et al. [21]	Body fat in %	1	**IG** 25	**CG** 25	-0.06 (-0.65 to 0.53)^b^	0.84	n.a.*	Low	Low
Yin et al. [22]		1	**IG** 25	**CG** 25	-0.06 (-0.65 to 0.53)	0.84	n.a.*	Low	Low
Hita -Contreras et al. [21]	SMMI	1	**IG** 25	**CG** 25	1.25 (0.64 to 1.86)^b^	< 0.0001	n.a.*	Low	Low
Yin et al. [22]		1	**IG** 25	**CG** 25	1.29 (0.68 to 1.90)	< 0.0001	n.a.*	Low	Low
Hita-Contreras et al. [21]	Grip strength	1	**IG** 25	**CG** 25	0.97 (-0.05 to 1.99)^b^	0.06	n.a.*	Low	Low
Yin et al. [22]		1	**IG** 25	**CG** 25	0.97 (-0.05 to 1.99)	0.06	n.a.*	Low	Low
Hita-Contreras et al. [21]	Gait speed in m/s	1	**IG** 25	**CG** 25	0.11 (0.02 to 0.20)^b^	0.02	n.a.*	Low	Low
Yin et al. [22]		1	**IG** 25	**CG** 25	0.11 (0.02 to 0.20)	0.02	n.a.*	Low	Low
**Protein supplementation**									
Yin et al. [22]	Body fat in %	2	**IG** 66	**CG** 68	-1.03 (-2.29 to 0.23)	0.11	37%	Moderate	Low
Hsu et al. [26]	Total body fat mass in kg	2	**IG** 45	**CG** 40	-0.35 (-2.41 to 1.72)	0.74	23%	Very low	Critically low
Hsu et al. [26]	Total muscle mass in kg	1	**IG** 12	**CG** 6	1.07 (-1.70 to 3.84)	0.45	n.a *	Very low	Critically low
Yin et al. [22]	SMMI	2	**IG** 66	**CG** 68	0.32 (-0.60 to 1.24)	0.49	86%	Low	Low
Hsu et al. [26]	Grip strength in kg	2	**IG** 66	**CG** 68	-0.11 (-2.02 to 1.80)	0.91	0%	Very low	Critically low
Yin et al. [22]		2	**IG** 66	**CG** 68	0.61 (-0.49 to 1.70)	0.28	0%	Moderate	Low
**Low-calorie + High-protein Intake**									
Hsu et al. [26]	Total body fat mass in kg	2	**IG** 63	**CG** 59	-0.82 (-1.34 to -0.30)	0.02	58%	Very low	Critically low
Hsu et al. [26]	Total muscle mass in kg	2	**IG** 63	**CG** 59	0.65 (-1.06 to 2.42)	0.46	0%	Very low	Critically low
Hsu et al. [26]	Grip strength in kg	2	**IG** 63	**CG** 59	0.68 (-1.06 to 2.42)	0.45	0%	Very low	Critically low
**Resistance training + Protein supplementation**									
Eglseer et al. [27••]	Total body fat mass in kg	2	**IG** 21	**CG** 23	-0.76 (-4.56 to 3.04)^d^	0.70	0%	Low	Moderate
Eglseer et al. [27••]	Total muscle mass in kg	2	**IG** 21	**CG** 23	-0.45 (-2.54 to 1.64)^d^	0.68	0%	Low	Moderate
**Mixed Training + Protein supplementation**									
Hita -Contreras et al. [21]	Body fat in %	1	**IG** 36	**CG** 34	-0.30 (-3.35 to 2.75)^b^	0.858	n.a.*	Low	Low
Yin et al. [22]		1	**IG** 36	**CG** 34	-0.30 (-2.54 to 1.94)	0.79	n.a.*	Low	Low
Hita -Contreras et al. [21]	Total body fat mass in kg	1	**IG** 36	**CG** 34	-0.20 (-3.26 to 2.86)^b^	0.90	n.a.*	Low	Low
Hita -Contreras et al. [21]	ASMM	1	**IG** 36_**!**_	**CG** 34_!_	-0.10 (-0.88 to 0.68)^a,b^	0.80	n.a.*	Low	Low
Yin et al. [22]		1	**IG** 36	**CG** 34	-0.10 (-1.12 to 0.92)	0.85	n.a.*	Low	Low
Hita -Contreras et al. [21]	SMMI	1	**IG** 36	**CG** 34	-0.02 (-0.49 to 0.44)^b^	0.92	n.a.*	Low	Low
Yin et al. [22]		1	**IG** 36	**CG** 34	-0.03 (-0.49 to 0.44)	0.92	n.a.*	Low	Low
Hita -Contreras et al. [21]	Grip strength in kg	1	**IG** 36	**CG** 34	0.70 (-2.34 to 3.74)^b^	0.65	n.a.*	Low	Low
Yin et al. [22]		1	**IG** 36	**CG** 34	0.70 (-1.53 to 2.93)	0.54	n.a.*	Low	Low
Hita -Contreras et al. [21]	Gait speed in m/s	1	**IG** 36	**CG** 34	0.00 (-0.13 to 0.13)^b^	1.00	n.a.*	Low	Low
Yin et al. [22]		1	**IG** 36	**CG** 34	0.00 (-0.09 to 0.09)	1.00	n.a.*	Low	Low
**Aerobic Training + Protein supplementation**									
Eglseer et al. [27••]	Total body fat mass in kg	1	**IG** 54	**CG** 50	-0.8 (-1.32 to 0.28)^e^	0.003	n.a.*	Low	Moderate
Eglseer et al. [27••]	Total muscle mass in kg	1	**IG** 54	**CG** 50	-0.8 (-1.32 to 0.28)^e^	0.003	n.a.*	Low	Moderate
**WB-EMS + Protein supplementation**									
Hita -Contreras et al. [21]	Body fat in %	2	**IG** 58	**CG** 59	-1.27 (-3.33 to 0.79)^b^	0.23	94%	Low	Low
Yin et al. [22]		2	**IG** 58	**CG** 59	-1.27 (-3.33 to 0.79)	0.23	94%	Very low	Low
Hita -Contreras et al. [21]	Total body fat mass in kg	1	**IG** 33	**CG** 34	-2.01 (-2.82 to -1.20)^b^	< 0.0001	n.a.*	Low	Low
Hita -Contreras et al. [21]	ASMM in kg	1	**IG** 33	**CG** 34	0.44 (0.20 to 0.68)^b^	0.0003	n.a.*	Low	Low
Yin et al. [22]		1	**IG** 33	**CG** 34	0.46 (0.22 to 0.70)	0.0002	n.a.*	Low	Low
Hita -Contreras et al. [21]	SMMI	2	**IG** 58	**CG** 59	0.72 (0.22 to 1.23)^b^	0.005	43%	Moderate	Low
Yin et al. [22]		2	**IG** 58	**CG** 59	1.18 (0.78 to 1.57)	< 0.0001	0%	Moderate	Low
Hita -Contreras et al. [21]	Grip strength in kg	2	**IG** 58	**CG** 59	1.10 (0.30 to 1.90)^b^	0.007	0%	Moderate	Low
Yin et al. [22]		2	**IG** 58	**CG** 59	1.31 (0.50 to 2.11)	0.001	0%	Moderate	Low
Hita -Contreras et al. [21]	Gait speed in m/s	2	**IG** 58	**CG** 59	0.04 (0.02 to 0.06)^b^	0.0001	0%	Moderate	Low
Yin et al. [22]		2	**IG** 58	**CG** 59	0.04 (0.02 to 0.06)	0.0001	0%	Moderate	Low

*IG* Intervention Group, *CG* Control Group, *CI* Confidence Interval

*na, not applicable

^a^IG was corrected from *n* = 6 to *n* = 36 according to the primary study by Kim et al. 2016, and the effect was newly calculated using the mean and SD according to [21]

^b^Effect size was calculated using RevMan 5.4

^c^Primary studies included in the reviews are partially overlapping

^d^Compared to resistance training alone

^e^Compared to aerobic training alone

Outcomes with partially significant improvements through resistance training were body fat in percent, total body fat mass and grip strength. Two out of the four reviews reported a significant decrease in the proportion of body fat, ranging from 1.53% (95% CI, -2.91; -0.15) [27••] to 2.67% (95% CI, -4.03; -1.32) [26] (CoE moderate and very low). Although the effects reported in the two other reviews were not significant, both showed a tendency in favour of resistance training (CoE moderate and low) [21, 22]. One review concluded that resistance training had a significant effect on total body fat mass with an average decrease of 3.28 kg (95% CI -5.63; -0.94) (CoE very low) [26]. A significant effect regarding grip strength was reported in two of four reviews, with average improvements ranging from 3.73 kg (95% CI, 2.60; 4.85) [27••] to 4.52 kg (95% CI, 1.88, 7.17) (CoE moderate and very low) [26]. Resistance training did not prove to be significantly effective for the outcome SMMI (CoE low and moderate) and total muscle mass (CoE very low).

### Effect of Aerobic Training

Total body fat mass was the only outcome for which significant improvement was observed through aerobic training. However, this significant effect was only measured in one out of the two reviews on total body fat mass with an average decrease of 5.2 kg (95% CI, -9.47 to -0.93) (CoE Very Low) [26].

### Effect of Mixed training

Measurements of the effect of mixed training on gait speed were reported in three reviews, and this was the only outcome for which a significant improvement was seen across all reviews with improvements ranging from 0.12 to 0.15 m/s (CoE very low to moderate) [21, 22, 26] see Table 3. Partially significant effects were measured for the outcomes of body fat in per cent and total body fat mass in kg. Regarding body fat in per cent, only one of three reviews cited a significant effect, reporting a decrease of 2.05% (95% CI, -3.50; -0.61) (CoE very low) [26], while for total body fat mass only one out of two reviews showed a significant improvement of 2.34 kg (95% CI, -4.26; -0.43) (CoE very low) [26].

### Effect of WB-EMS

SMMI and gait speed were the only outcomes that showed significant improvements as a result of WB-EMS. This was seen in all reviews that measured the respective outcomes. The effects of WB-EMS on SMMI and gait speed were measured in two reviews each [21, 22]. Regarding SMMI, both reviews reported significant improvements that ranged from 1.25 (95% CI, 0.64; 1.86) [21] to 1.29 kg (95% CI, 0.68; 1.90) [22] (CoE low), while gait speed improved by 0.11 m/s (95% CI, 0.02; 0.20) (CoE low) [21, 22]. WB-EMS was not reported as showing a significant improvement in any review of measurements of outcomes on body fat in percent (CoE low) or grip strength (CoE low).

## Nutrition Interventions

### Effect of Protein supplementation

Two reviews analysed the effect of protein supplementation [22, 26]. No significant improvement was reported in the studied outcomes, i.e. body fat in percent, total body fat mass in kg, total muscle mass in kg, SMMI, or grip strength in kg (CoE very low to moderate).

### Effect of Low-calorie and High-Protein Intake Diets

One review analysed the effects of a low-calorie diet together with high protein intake [26]. Significant improvement in terms of total body fat mass in kg was observed, leading to a decrease in weight of 0.82 kg (95% CI, -1.34; -0.30) (CoE very low). No significant effect was measured for other outcomes.

## Exercise and Nutrition Interventions

### Effect of Resistance Training and Protein supplementation

The effects of resistance training together with protein supplementation were analysed with respect to total body fat mass in kg and total muscle mass compared to only resistance training in the review by Eglseer et a. Both body fat mass in kg and muscle mass in kg did not significantly improve (CoE low) [27••].

### Effect of Aerobic Training and Protein supplementation

The combination of aerobic training and protein supplementation was analysed in the review by Eglseer et al. regarding total fat and muscle mass. This type of intervention was not significantly effective for both outcomes compared to performing aerobic training alone (CoE low) [27••].

### Effect of Mixed Training and Protein supplementation

The combination of mixed training and protein supplementation did not provide a significant extra benefit compared to mixed training alone. This was seen for the outcomes body fat in per cent, total body fat mass in kg, ASMM, SMMI, grip strength in kg, or gait speed in m/s (CoE low) [21, 22].

### WB-EMS and Protein supplementation

WB-EMS combined with supplementation significantly improved total body fat mass in kg, ASMM, SMMI, grip strength and gait speed. The effect on total body fat mass was only analysed by Hita-Contreras et al. [22], who reported a weight loss of 2.01 kg (95% CI, -2.82 to -1.20) (CoE low). ASMM improved by 0.44 kg (95% CI, 0.20,0.68) to 0.46 kg (95% CI, 0.22; 0.70) (CoE low), SMMI improved by 0.72 (95% CI, 0.22; 1.23) to 1.18 kg (95% CI, 0.78; 1.57) (CoE moderate), and grip strength improved by 1.10 kg (95% CI, 0.30; 1.90) to 1.31 kg (95% CI, 0.50; 2.11) (CoE moderate) [21, 22]. WB-EMS was reported as improving gait speed by 0.04 m/s (95% CI, 0.02; 0.06) (CoE moderate) in two reviews [21, 22].

### Adverse events

None of the included reviews gave statements on any adverse events or injuries that might have occurred during the trials.

### GRADE

The overall confidence in the evidence across all outcomes and interventions varied from very low to moderate (Table 3, Online Resource 2). When inspecting the ratings of individual criteria that served as the basis for assessing the certainty of the evidence (risk of bias, publication bias, imprecision, inconsistency and indirectness), we can see that the number of primary studies included in the reviews clearly had a profound influence on the GRADE rating. As all of the reviews regarding the outcomes for the interventions of aerobic training, WB-EMS, low-calorie combined with high-protein intake, resistance training, aerobic training combined with protein supplementation, and mixed exercise combined with increased protein intake included only a single primary study, the imprecision was automatically rated as very serious. Further influential factors regarding imprecision were the small sample sizes, wide CI and the overall effect being insignificant. In contrast, the low number of included primary studies resulted in most of the outcomes showing no serious inconsistencies, but the serious inconsistencies noted were mainly due to a partial overlap of the CIs and considerable heterogeneity. Hsu et al. [26] was the only study that was rated as having a serious risk of bias as well as a publication bias. This was due to the lack of allocation concealment, blinding and application of Egger’s test. All other reviews and the respective outcomes that they measured had no serious risk of bias and publication bias. Regarding indirectness, all reviews included primary studies in accordance with their inclusion and exclusion criteria,therefore, the indirectness was rated as low for all outcomes across all interventions.

An overview of the results and their certainty in evidence can be found in Table 3 and Online Resource 2.

## Discussion

In this umbrella review, which included four systematic reviews that each included between 30 to 225 participants per included outcome, we provide comprehensive insights into the exercise and nutrition interventions for the treatment of SO that have been studied to date, as well as their certainties of the evidence.

Resistance training was the most frequently studied intervention. This training improved gait speed and muscle strength in persons with sarcopenic obesity, but contradictory results were reported for the outcomes on body fat, SMMI and grip strength. Interestingly, gait speed was significantly improved in four of the five interventions that measured gait speed (resistance training, mixed training, WB-EMS, mixed training + protein supplementation, WB-EMS + protein supplementation). Nevertheless, it must be noted that none of these interventions led to a clinically relevant improvement higher than 0.5 m/s [28]. Mixed exercise (combining resistance with aerobic training) was also one of the most frequently studied interventions, but contradictory results were also found for improving body composition and grip strength, with the certainty of evidence also ranging from moderate to very low.

Although the effect of resistance training for people with SO yielded partially contradictory results, its positive effects on sarcopenia and obesity as separate conditions have been verified in several studies [29•, 30]. A recent systematic review and meta-analysis which included 114 trials reported that resistance training significantly decreases the percentage of body fat and overall fat mass in overweight or obese adults of all ages [29•]. Additionally, an umbrella review conducted in 2019 provides high-quality evidence that resistance training in older adults with diagnosed sarcopenia significantly enhances muscle strength, mass and performance. However, the same review also stated that a low certainty of evidence exists against using a combination of resistance training and nutritional supplementation, such as increased protein intake [30]. With a low certainty of evidence, our umbrella review results reveal that protein supplementation combined with resistance training and protein supplementation combined with aerobic training and mixed training are not significantly effective interventions for persons with SO. Furthermore, our results on protein supplementation alone presented no significant improvement for any of the measured outcomes. Even in combination with a low-calorie intake, protein supplementation only improved the outcome of total body fat mass. To date, the effect of protein supplementation in persons with SO is still to be explored. Due to the low certainty of evidence for protein supplementation in persons with SO, future researchers may change these previous results. However, contradictory results are also reported regarding protein supplementation with or without exercise in persons with sarcopenia [31], which supports our results.

The results of this umbrella review show that WB-EMS improved muscle mass, muscle strength and physical function in persons with SO. Similar results can be seen in a recent systematic review measuring the effect of WB-EMS in older adults, where 12 out of the 13 included primary studies with participants were either diagnosed with obesity, SO, or at risk of sarcopenia. The results show a significant improvement in gait speed, hand grip strength, lower limb strength and ASMMI [18]. Despite the positive effects on sarcopenic parameters, the wearable device described is extremely expensive, and unsupervised private WB-EMS application has previously been associated with adverse effects such as musculoskeletal injuries [32]. For these reasons, we advise against using WB-EMS unless its use is strictly monitored by a professional.

Prior studies have proven that there are significant differences in body composition and strength regarding gender, with males being at an advantage [33–35]. As this systematic review did not set a limitation on gender, there is a possibility that the effects on the previously mentioned outcomes are distorted. Therefore, it is recommended that future primary studies report mean differences in percentage or perform gender specific analyses.

Our umbrella review highlights the need to improve the number and quality of RCTs as well as systematic reviews in this field of research. Despite our rigorous search strategy, we were only able to find a small number of systematic reviews. The number of included primary studies supporting the effects was also very low across all interventions and outcomes. During the study, we realized that especially the effects of nutrition interventions as well as the combination of exercise and nutrition interventions have only been vaguely explored with regard to SO. Therefore, in future, studies of high quality on the effect of hypocaloric diets with/without exercise and increased protein intake are needed. The small number of included primary studies also affected the certainty of the evidence. In most of the studied interventions and outcomes, we have only limited confidence in the effect estimates, meaning that the true effect may change if more high-quality studies are conducted. Moreover, the differences in AMSTAR ratings ranged from moderate to critically low, showing that the methodological quality of the individual systematic reviews additionally impacts the certainty of the evidence and leads to variance in certainty. This, in combination with the contradictory results, did not allow us to make specific recommendations regarding the most effective intervention for persons with SO. Moreover, the ideal duration, dose, intensity, or amount of exercise, as well as nutrition interventions, still need to be explored. As the consensus on the definition and diagnosis of sarcopenic obesity has only recently been published, the variety of criteria and definitions of sarcopenia found in research is still great. This can also be seen in the characteristics of the included reviews of this study and might be an explanation of the lack of significant results. However, it can be expected that the consensus leads to inclusion of a more homogenous population of future studies [8••]. In turn, this will enhance the comparability of research results and facilitate the process of making specific recommendations for persons with SO.

Nevertheless, this review also has some limitations. Firstly, the literature search of this umbrella review was constricted to the last five years. However, as none of the included systematic reviews had set a time limit, it was presumed that the systematic reviews included all relevant primary studies conducted on the effectiveness of interventions for persons with SO so far. Secondly, due to the design of the umbrella review, we did not consider data from the primary studies and trusted that the authors of the systematic reviews had extracted and pooled the data from the primary studies to the best of their abilities. Nevertheless, we found indications that errors had been made by the authors conducting the systematic reviews. For example, it came to our attention that both Hita-Contreras et al. [21] and Yin et al. [22] analysed the effects of mixed exercise combined with protein supplementation, but resulted in slightly different results for each of the outcomes concerning this intervention, although the same number of primary studies and the same number of participants were included. Despite assessing the methodology of each review, it was not always possible to explain the observed differences in effects and CIs. Moreover,, the systematic reviews included primary studies which used different definitions of SO. This might have led to contradictory results and, furthermore, decreases the comparability of the results. Furthermore, it must be taken into consideration that this umbrella review only included data from reviews which had performed meta-analyses.

## Conclusion

We found only four systematic reviews and meta-analyses investigating nutrition and exercise interventions in persons with SO. Resistance training was the most frequently studied intervention and showed the most promising results for improving muscle strength and physical performance. Regarding nutrition interventions, such as protein supplementation with or without calorie restriction and/or exercise, little evidence of effectiveness exists for this target group. Although the data are largely lacking, conducting further high-quality studies are likely to change these results. Due to the partly substandard methodology of the included reviews and the low number of primary studies, we were unable to identify an ideal intervention for persons with SO. Our umbrella review provides clear evidence that the effects of nutrition and exercise interventions in persons with SO requires further intensive study to provide specific recommendations.

## Supplementary Information

Below is the link to the electronic supplementary material.

Supplementary file1 (DOCX 16.9 KB)

Supplementary file2 (DOCX 40.5 KB)
