# Supplementary material for: Effects of Nutrition and Exercise Interventions on Persons with Sarcopenic Obesity: An Umbrella Review of Meta-Analyses of Randomised Controlled Trials
Source: Curr Obes Rep. 2023 May 30;12(3):250–63. doi: 10.1007/s13679-023-00509-0 (PMC10482763; doi:10.1007/s13679-023-00509-0)
Supplement: Supplementary file 1 — Supplementary file1 (DOCX 16.9 KB) [file 13679_2023_509_MOESM1_ESM.docx]

**Supplementary Table 1:** Search strategy and number of results regarding the different databases

| **Database** | **Search strategy** | **Results** |
| --- | --- | --- |
| PubMed | ((((((((" Exercise" [Mesh]) OR (train*)) OR (physical activity) OR (exercise)) OR ("Diet, Food, and Nutrition"[Mesh])) OR ("Nutrition Therapy" [Mesh])) OR (diet)) OR (nutr*)) OR (energy restriction)) AND ((((("Sarcopenia"[Mesh]) AND ("Obesity" [Mesh])) OR (obese sarcopen*)) OR (sarcobesity)) OR (sarcopenic obes*)) Filters: Meta-Analysis, Systematic Review, in the last 5 years, Middle Aged + Aged: 45+ years. | 21 |
| EMBASE via OVID | (((obesity[MeSH Terms]) AND (sarcopenia[MeSH Terms])) OR (obese sarcopen*) OR (sarcobesity) OR (sarcopenic obes*)) AND ((exercise[MeSH Terms]) OR (train*) OR physical activity (MeSH) OR (physical activity) OR (exercise)) OR ((diet Therapy[MeSH Terms]) OR (diet) OR (nutr*) OR (energy restriction)) Filters: Meta-Analyses/Reviews/Systematic Reviews, in the last 5 years, Aged 18 + | 28 |
| Cochrane Database of Systematic Reviews via OVID | (sarcopenic obes*)) AND (train*) OR (physical activity) OR (exercise)) OR (diet) OR (nutr*) OR (energy restriction)) | 2 |
