# Supplementary material for: Effects of Nutrition and Exercise Interventions on Persons with Sarcopenic Obesity: An Umbrella Review of Meta-Analyses of Randomised Controlled Trials
Source: Curr Obes Rep. 2023 May 30;12(3):250–63. doi: 10.1007/s13679-023-00509-0 (PMC10482763; doi:10.1007/s13679-023-00509-0)
Supplement: Supplementary file 2 — Supplementary file2 (DOCX 40.5 KB) [file 13679_2023_509_MOESM2_ESM.docx]

**Supplementary Table 2**: GRADE rating and extracted data from reviews studying interventions regarding exercise, nutrition and a combination of exercise and nutrition

| **Intervention** | **A** | **Metric** | **No** | **RoB** | **Inconsistency** | **Indirectness** | **Imprecision** | **Publication Bias** | **Sample** | | **Effect (95% of CI)** | ***p*** | ***I*^2^ %** | **Confidence in Evidence** |
| --- | --- | --- | --- | --- | --- | --- | --- | --- | --- | --- | --- | --- | --- | --- |
| **Resistance Training** | | | | | | | | | | | | | | |
| Body fat in % | Eg | MD | 4 | No serious risk of bias_5_ | Not serious_9, 11_ | Not serious | Serious_16, 35_ | Not serious_24_ | IG 104 | CG 94 | -1.53 (-2.91 to -0.15) | 0.03 | 28% | Moderate |
|  | H-C | MD | 3 | No serious risk of bias_1_ | Not serious_8_ | Not serious | Very serious_12, 15, 16, 31_ | Not serious_23_ | IG 102 | CG 96 | -0.56 (-1.46 to 0.34)_★_ | 0.22 | 0 % | Low |
|  | Hs | MD | 5 | Serious risk of bias_3_ | Not serious_9, 11_ | Not serious | Serious_12, 36_ | Serious_22_ | IG 121 | CG 104 | -2.67 (-4.03 to -1.32) | <0.05 | 17% | Very low |
|  | Yin | MD | 3 | No serious risk of bias_21_ | Not serious_a, 9, 32_ | Not serious | Serious_26,_ _31_ | Not serious_25_ | IG 117 | CG 102 | -1.82 (-4.29 to 0.65) | 0.15 | 66% | Moderate |
| Total body fat mass in kg | H-C | MD | 2 | No serious risk of bias_1_ | Not serious_8_ | Not serious | Very serious_12,_ _13_ | Not serious_23_ | IG 33 | CG 32 | -1.45 (-5.04 to 2.14)_★_ | 0.43 | 0% | Low |
|  | Hs | MD | 3 | Serious risk of bias_3_ | Not serious_8_ | Not serious | Serious_12,_ _26_ | Serious_22_ | IG 58 | CG 53 | -3.28 (-5.63 to-0.94) | 0.006 | 0% | Very low |
| Total muscle mass in kg | Eg | MD | 3 | No serious risk of bias_5_ | Serious_11,_ _27_ | Not serious | Very serious_12,_ _15_ | Not serious_24_ | IG 73 | CG 63 | -0.01 (-0.98 to 0.96) | 0.99 | 11% | Very low |
|  | Hs | MD | 4 | Serious risk of bias_3_ | Not serious_8_ | Not serious | Very serious_12,_ _15_ | Serious_22_ | IG 88 | CG 73 | 0.36 (-0.96 to 1.68) | 0.59 | 0% | Very low |
| SMMI | H-C | SMD | 1 | No serious risk of bias_1_ | Not serious_7_ | Not serious | Very serious_7,_ _12,_ _15_ | Not serious_23_ | IG 15 | CG 15 | 0.18 (-0.54 to 0.89)_★_ | 0.63 | n.a.* | Low |
|  | Yin | SMD | 2 | No serious risk of bias_21_ | Not serious_8_ | Not serious | Serious_12,_ _14,_ _20_ | Not serious_25_ | IG 48 | CG 38 | 0.28 (-0.15 to 0.71) | 0.20 | 0% | Moderate |
| Grip strength in kg | Eg | MD | 3 | No serious risk of bias_5_ | Not serious_8_ | Not serious | Serious_12,_ _33_ | Not serious_24_ | IG 73 | CG 63 | 3.73 (2.60 to 4.85) | <0.0001 | 0% | Moderate |
|  | H-C | MD | 1 | No serious risk of bias_1_ | Not serious_7_ | Not serious | Very serious_7,_ _12,_ _15,_ _16_ | Not serious_23_ | IG 15 | CG 15 | 6.00 (-1.68 to 13.68)_★_ | 0.13 | n.a.* | Low |
|  | Hs | MD | 3 | Serious risk of bias_3_ | Not serious_8_ | Not serious | Serious_7,_ _16,_ _33_ | Serious_22_ | IG 73 | CG 67 | 4.52 (1.88 to 7.17) | 0.0008 | 0% | Very low |
|  | Yin | MD | 2 | No serious risk of bias_21_ | Not serious_9,_ _11_ | Not serious | Serious_12,_ _20,_ _37_ | Not serious_25_ | IG 48 | CG 38 | 2.88 (-0.88 to 6.64) | 0.13 | 46% | Moderate |
| Lower/leg extremity strength in kg | Eg | MD | 1 | No serious risk of bias_5_ | Not serious_7_ | Not serious | Very serious_7,_ _12,_ _16,_ | Not serious_24_ | IG 14 | CG 14 | 9.97 (4.43 to 15.51) | 0.0004 | n.a.* | Low |
| Gait speed in m/s | Eg | MD | 3 | No serious risk of bias_5_ | Serious_10,_ _e_ | Not serious | Serious_12,_ _33_ | Not serious_24_ | IG 72 | CG 62 | 0.17 (0.01 to 0.34) | 0.04 | 85% | Low |
|  | Hs | MD | 3 | Serious risk of bias_3_ | Serious_10,_ _e_ | Not serious | Serious_12,_ _14,_ _33_ | Serious_22_ | IG 69 | CG 55 | 0.23 (0.00 to 0.46) | 0.05 | 91 % | Very low |
|  | Yin | MD | 1 | No serious risk of bias_21_ | Not serious_7_ | Not serious | Very serious_7,_ _12,_ _14,_ _19_ | Not serious_25_ | IG 33 | CG 23 | 0.14 (0.05 to 0.23) | 0.002 | n.a.* | Low |
| **Aerobic Training** | | | | | | | | | | | | | | |
| Body fat in % | H-C | MD | 1 | No serious risk of bias_1_ | Not serious_7_ | Not serious | Very serious_7,_ _15,_ _16_ | Not serious_23_ | **IG** 15 | **CG** 15 | -1.70 (-5.90 to 2.50)_★_ | 0.43 | n.a* | Low |
|  | Hs | MD | 1 | Serious risk of bias_3_ | Not serious_7_ | Not serious | Very serious_7,_ _16,_ _17,_ _20_ | Serious_22_ | **IG** 15 | **CG** 15 | -1.50 (-4.83 to 1.83) | 0.38 | n.a* | Very low |
|  | Yin | MD | 1 | No serious risk of bias_21_ | Not serious_7_ | Not serious | Very serious_7,_ _16,_ _17,_ _20_ | Not serious_25_ | **IG** 15 | **CG** 15 | -1.70 (-4.99 to 1.59) | 0.31 | n.a* | Low |
| Total body fat mass in kg | H-C | MD | 1 | No serious risk of bias_1_ | Not serious_7_ | Not serious | Very serious_7,_ _15,_ _17_ | Not serious_23_ | **IG** 15 | **CG** 15 | -1.50 (-6.99 to 3.99)_★_ | 0.59 | n.a.* | Low |
|  | Hs | MD | 1 | Serious risk of bias_3_ | Not serious_7_ | Not serious | Very Serious_7,_ _16,_ _17_ | Serious_22_ | **IG** 15 | **CG** 15 | -5.20 (-9.47 to -0.93) | 0.02 | n.a.* | Very low |
| Total muscle mass in kg | Hs | MD | 1 | Serious risk of bias_3_ | Not serious_7_ | Not serious | Very serious_7,_ _16,_ _17,_ _20_ | Serious_22_ | **IG 15** | **CG 15** | -1.00 (-3.51 to 1.51) | 0.44 | n.a.* | Very low |
| SMMI | H-C | SMD | 1 | No serious risk of bias_1_ | Not serious_7_ | Not serious | Very serious_7,_ _12,_ _15_ | Not serious_23_ | **IG** 15 | **CG** 15 | 0.26 (-0.46 to 0.98)_★_ | 0.48 | n.a.* | Low |
|  | Yin | SMD | 1 | No serious risk of bias_21_ | Not serious_7_ | Not serious | Very serious_7,_ _12,_ _14,_ _20_ | Not serious_25_ | **IG** 15 | **CG** 15 | 0.33 (-0.39 to 1.05) | 0.37 | n.a.* | Low |
| Grip strength in kg | H-C | MD | 1 | No serious risk of bias_1_ | Not serious_7_ | Not serious | Very serious_7,_ _12,_ _15_ | Not serious_23_ | **IG** 15 | **CG** 15 | -0.40 (-8.08 to 7.28)_★_ | 0.92 | n.a.* | Low |
|  | Hs | MD | 1 | Serious risk of bias_3_ | Not serious_7_ | Not serious | Very serious_7,_ _12,_ _15_ | Serious_22_ | **IG** 15 | **CG** 15 | -0.50 (-6.22 to 5.22) | 0.86 | n.a.* | Very low |
|  | Yin | MD | 1 | No serious risk of bias_21_ | Not serious_7_ | Not serious | Very serious_7,_ _12,_ _15,_ _16_ | Not serious_25_ | **IG** 15 | **CG** 15 | -0.40 (-6.35 to 5.55) | 0.90 | n.a.* | Low |
| **Mixed Training** | | | | | | | | | | | | | | |
| Body fat in % | H-C | MD | 3 | No serious risk of bias_1_ | Not serious_8_ | Not serious | Very serious_12,_ _15_ | Not serious_23_ | **IG** 75 | **CG** 74 | -1.63 (-3.53 to 0.27)_★_ | 0.09 | 0% | Low |
|  | Hs | MD | 3 | Serious risk of bias_3_ | Not serious_8_ | Not serious | Serious_12,_ _13_ | Serious_22_ | **IG** 74 | **CG** 74 | -2.05 (-3.50 to -0.61) | 0.005 | 0% | Very low |
|  | Yin | MD | 3 | No serious risk of bias_21_ | Not serious_9,_ _11,_ _19,_ _32_ | Not serious | Serious_16,_ _26_ | Not serious_25_ | **IG** 74 | **CG** 74 | -1.63 (-3.30 to 0.03) | 0.05 | 23% | Moderate |
| Total body fat mass in kg | H-C | MD | 2 | No serious risk of bias_1_ | Not serious_8_ | Not serious | Very serious_12,_ _15_ | Not serious_23_ | **IG** 50 | **CG** 49 | -0.40 (-3.08 to 2.27)_★_ | 0.77 | 0% | Low |
|  | Hs | MD | 2 | Serious risk of bias_3_ | Serious_a,_ _10_ | Not serious | Serious_12,_ _16,_ _18_ | Serious_22_ | **IG** 49 | **CG** 49 | -2.34 (-4.26 to -0.43) | 0.02 | 66% | Very low |
| Total muscle mass in kg | Hs | MD | 1 | Serious risk of bias_3_ | Not serious_7_ | Not serious | Very serious_7,_ _12,_ _15_ | Serious_22_ | **IG 15** | **CG 15** | 0.20 (-2.48 to 2.88) | 0.88 | n.a.* | Very low |
| ASMM in kg | H-C | MD | 2 | No serious risk of bias_1_ | Not serious_8_ | Not serious | Very serious_12,_ _15_ | Not serious_23_ | **IG** 60 | **CG** 59 | 0.22 (-0.69 to 1.13) | 0.63 | 0% | Low |
|  | Yin | MD | 2 | No serious risk of bias_21_ | Not serious_8_ | Not serious | Serious_12,_ _20_ | Not serious_25_ | **IG** 59 | **CG** 59 | 0.25 (-0.47 to 0.98) | 0.50 | 0% | Moderate |
| SMMI | H-C | SMD | 2 | No serious risk of bias_1_ | Not serious_9,_ _11_ | Not serious | Very serious_12,_ _15_ | Not serious_23_ | **IG** 50 | **CG** 49 | -0.03 (-0.56 to 0.51)_★_ | 0.96 | 38% | Low |
|  | Yin | SMD | 2 | No serious risk of bias_21_ | Not serious_9,_ _e_ | Not serious | Serious_12,_ _20_ | Not serious_25_ | **IG** 49 | **CG** 49 | 0.02 (-0.61 to 0.66) | 0.94 | 55% | Moderate |
| Grip strength | H-C | MD | 3 | No serious risk of bias_1_ | Not serious_a,_ _9_ | Not serious | Serious_12,_ _16,_ _23_ | Not serious_23_ | **IG** 75 | **CG** 74 | 1.86 (-1.04 to 4.75)_★_ | 0.21 | 59% | Moderate |
|  | Hs | MD | 3 | Serious risk of bias_3_ | Serious_10,_ _e_ | Not serious | Very serious_20,_ _26_ | Serious_22_ | **IG** 74 | **CG** 74 | 2.33 (-1.63 to 6.30) | 0.25 | 88% | Very low |
|  | Yin | MD | 3 | No serious risk of bias_21_ | Serious_10,_ _e_ | Not serious | Serious_20,_ _26,_ _37_ | Not serious_25_ | **IG** 74 | **CG** 74 | 1.71 (-1.25 to 4.68) | 0.26 | 78% | Low |
| Gait speed in m/s | H-C | MD | 2 | No serious risk of bias_1_ | Not serious_8_ | Not serious | Serious_12,_ _19,_ _30_ | Not serious_23_ | **IG** 60 | **CG** 59 | 0.12 (0.02 to 0.22)_★_ | 0.02 | 0% | Moderate |
|  | Hs | MD | 2 | Serious risk of bias_3_ | Not serious_a,_ _9_ | Not serious | Serious_12,_ _14,_ _18_ | Serious_22_ | **IG** 59 | **CG** 59 | 0.15 (0.04 to 0.26) | 0.006 | 51% | Very low |
|  | Yin | MD | 2 | No serious risk of bias_21_ | Not serious_9,_ _11_ | Not serious | Serious_12,_ _14,_ _19_ | Not serious_25_ | **IG** 59 | **CG** 59 | 0.14 (0.05 to 0.23) | 0.002 | 27% | Moderate |
| **WB-EMS** | | | | | | | | | | | | | | |
| Body fat in % | H-C | MD | 1 | No serious risk of bias_1_ | Not serious_7_ | Not serious | Very serious_7,_ _15,_ _17_ | Not serious_23_ | **IG** 25 | **CG** 25 | -0.06 (-0.65 to 0.53)_★_ | 0.84 | n.a.* | Low |
|  | Yin | MD | 1 | No serious risk of bias_21_ | Not serious_7_ | Not serious | Very serious_7,_ _12,_ _20_ | Not serious_25_ | **IG** 25 | **CG** 25 | -0.06 (-0.65 to 0.53) | 0.84 | n.a.* | Low |
| SMMI | H-C | SMD | 1 | No serious risk of bias_1_ | Not serious_7_ | Not serious | Very serious_7,_ _14,_ _17,_ _19_ | Not serious_23_ | **IG** 25 | **CG** 25 | 1.25 (0.64 to 1.86)_★_ | <0.0001 | n.a.* | Low |
|  | Yin | MD | 1 | No serious risk of bias_21_ | Not serious_7_ | Not serious | Very serious_7,_ _12,_ _14,_ _19_ | Not serious_25_ | **IG** 25 | **CG** 25 | 1.29 (0.68 to 1.90) | <0.0001 | n.a.* | Low |
| Grip strength | H-C | MD | 1 | No serious risk of bias_1_ | Not serious_7_ | Not serious | Very serious _7,_ _12,_ _15_ | Not serious_23_ | **IG** 25 | **CG** 25 | 0.97 (-0.05 to 1.99)_★_ | 0.06 | n.a.* | Low |
|  | Yin | MD | 1 | No serious risk of bias_21_ | Not serious_7_ | Not serious | Very serious_7,12,_ _20_ | Not serious_25_ | **IG** 25 | **CG** 25 | 0.97 (-0.05 to 1.99) | 0.06 | n.a.* | Low |
| Gait speed in m/s | H-C | MD | 1 | No serious risk of bias_1_ | Not serious_7_ | Not serious | Very serious_7,_ _17,_ _19_ | Not serious_23_ | **IG** 25 | **CG** 25 | 0.11 (0.02 to 0.20)_★_ | 0.02 | n.a.* | Low |
|  | Yin | MD | 1 | No serious risk of bias_21_ | Not serious_7_ | Not serious | Very serious_7,_ _12,_ _19_ | Not serious_25_ | **IG** 25 | **CG** 25 | 0.11 (0.02 to 0.20) | 0.02 | n.a.* | Low |
| **Protein Supplementation** | | | | | | | | | | | | | | |
| Body fat in % | Yin | MD | 2 | No serious risk of bias_21_ | Not serious_9,_ _11_ | Not serious | Serious_7,_ _12,_ _20_ | Not serious_25_ | **IG** 66 | **CG** 68 | -1.03 (-2.29 to 0.23) | 0.11 | 37% | Moderate |
| Total body fat mass in kg | Hs | MD | 2 | Serious risk of bias_3_ | Not serious_9,_ _11_ | Not serious | Very serious _7,_ _15,_ _16,_ _1_ | Serious_22_ | **IG** 45 | **CG** 40 | -0.35 (-2.41 to 1.72) | 0.74 | 23% | Very low |
| Total muscle mass in kg | Hs | MD | 1 | Serious risk of bias_3_ | Not serious_7_ | Not serious | Very serious_7,_ _15,_ _17_ | Serious_22_ | **IG** 12 | **CG** 6 | 1.07 (-1.70 to 3.84) | 0.45 | n.a | Very low |
| SMMI | Yin | SMD | 2 | No serious risk of bias_21_ | Serious_10,_ _e_ | Not serious | Serious_18,_ _29_ | Not serious_25_ | **IG** 66 | **CG** 68 | 0.32 (-0.60 to 1.24) | 0.49 | 86% | Low |
| Grip strength in kg | Hs | MD | 2 | Serious risk of bias_3_ | Not serious_8_ | Not serious | Very serious_15,_ _17_ | Serious_22_ | **IG** 66 | **CG** 68 | -0.11 (-2.02 to 1.80) | 0.91 | 0% | Very low |
|  | Yin | MD | 2 | No serious risk of bias_21_ | Not serious_8_ | Not serious | Serious _12,_ _16,_ _20_ | Not serious_25_ | **IG** 66 | **CG** 68 | 0.61 (-0.49 to 1.70) | 0.28 | 0% | Moderate |
| **Low-calorie + High-protein Intake** | | | | | | | | | | | | | | |
| Total body fat mass in kg | Hs | MD | 2 | Serious risk of bias_3_ | Serious_a,_ _27_ | Not serious | Very serious_7,_ _15,_ _16,_ _17_ | Serious_22_ | **IG** 63 | **CG** 59 | -0.82 (-1.34 to -0.30) | 0.02 | 58% | Very low |
| Total muscle mass in kg | Hs | MD | 2 | Serious risk of bias_3_ | Not serious_8_ | Not serious | Very serious_12,_ _15_ | Serious_22_ | **IG** 63 | **CG** 59 | 0.65 (-1.06 to 2.42) | 0.46 | 0% | Very low |
| Grip strength in kg | Hs | MD | 2 | Serious risk of bias_3_ | Not serious_8_ | Not serious | Very serious_15,_ _17_ | Serious_22_ | **IG** 63 | **CG** 59 | 0.68 (-1.06 to 2.42) | 0.45 | 0% | Very low |
| **Resistance Training + Protein Supplementation** | | | | | | | | | | | | | | |
| Total body fat mass in kg | Eg | MD | 2 | No serious risk of bias_5_ | Not serious_8_ | Not serious | Very serious_16,_ _17,_ _20_ | Not serious_24_ | **IG** 21 | **CG** 23 | -0.76 (-4.56 to 3.04) | 0.70 | 0% | Low |
| Total muscle mass in kg | Eg | MD | 2 | No serious risk of bias_5_ | Not serious_8_ | Not serious | Very serious_12,_ _15,_ _16_ | Not serious_24_ | **IG** 21 | **CG** 23 | -0.45 (-2.54 to 1.64) | 0.68 | 0% | Low |
| **Mixed Training + Protein Supplementation** | | | | | | | | | | | | | | |
| Body fat in % | H-C | MD | 1 | No serious risk of bias_1_ | Not serious_7_ | Not serious | Very serious_7,_ _12,_ _15_ | Not serious_23_ | **IG** 36 | **CG** 34 | -0.30 (-3.35 to 2.75)_★_ | 0.858 | n.a.* | Low |
|  | Yin | MD | 1 | No serious risk of bias_21_ | Not serious_7_ | Not serious | Very Serious_7,_ _12,_ _20_ | Not serious_25_ | **IG** 36 | **CG** 34 | -0.30 (-2.54 to 1.94) | 0.79 | n.a.* | Low |
| Total body fat mass in kg | H-C | MD | 1 | No serious risk of bias_1_ | Not serious_7_ | Not serious | Very serious _7,_ _12,_ _15_ | Not serious_23_ | **IG** 36 | **CG** 34 | -0.20 (-3.26 to 2.86)_★_ | 0.90 | n.a.* | Low |
| ASMM | H-C | MD | 1 | No serious risk of bias_1_ | Not serious_7_ | Not serious | Very serious_7,_ _12,_ _15_ | Not serious_23_ | **IG** 36**_!_** | **CG** 34**_!_** | -0.10 (-0.88 to 0.68)**_!_**_★_ | 0.80 | n.a.* | Low |
|  | Yin | MD | 1 | No serious risk of bias_21_ | Not serious_7_ | Not serious | Very serious_7,_ _12,_ _20_ | Not serious_25_ | **IG** 36 | **CG** 34 | -0.10 (-1.12 to 0.92) | 0.85 | n.a.* | Low |
| SMMI | H-C | SMD | 1 | No serious risk of bias_1_ | Not serious_7_ | Not serious | Very serious_7,_ _12,_ _15_ | Not serious_23_ | **IG** 36 | **CG** 34 | -0.02 (-0.49 to 0.44)_★_ | 0.92 | n.a.* | Low |
|  | Yin | SMD | 1 | No serious risk of bias_21_ | Not serious_7_ | Not serious | Very serious_7,_ _12,_ _20_ | Not serious_25_ | **IG** 36 | **CG** 34 | -0.03 (-0.49 to 0.44) | 0.92 | n.a.* | Low |
| Grip strength in kg | H-C | MD | 1 | No serious risk of bias_1_ | Not serious_7_ | Not serious | Very serious_7,_ _15,_ _17_ | Not serious_23_ | **IG** 36 | **CG** 34 | 0.70 (-2.34 to 3.74)_★_ | 0.65 | n.a.* | Low |
|  | Yin | MD | 1 | No serious risk of bias_21_ | Not serious_7_ | Not serious | Very serious_7,12,_ _16,_ _20_ | Not serious_25_ | **IG** 36 | **CG** 34 | 0.70 (-1.53 to 2.93) | 0.54 | n.a.* | Low |
| Gait speed in m/s | H-C | MD | 1 | No serious risk of bias_1_ | Not serious_7_ | Not serious | Very serious_7,_ _14,_ _15,_ _17_ | Not serious_23_ | **IG** 36 | **CG** 34 | 0.00 (-0.13 to 0.13)_★_ | 1.00 | n.a.* | Low |
|  | Yin | MD | 1 | No serious risk of bias_21_ | Not serious_7_ | Not serious | Very serious_7,_ _12,_ _14,_ _15_ | Not serious_25_ | **IG** 36 | **CG** 34 | 0.00 (-0.09 to 0.09) | 1.00 | n.a.* | Low |
| **Aerobic Training + Protein Supplementation** | | | | | | | | | | | | | | |
| Total body fat mass in kg | Eg | MD | 1 | No serious risk of bias_5_ | Not serious_7_ | Not serious | Very Serious_7,_ _12,_ _14,_ _19_ | Not serious_24_ | **IG** 54 | **CG** 50 | -0.8 (-1.32 to 0.28) | 0.003 | n.a.* | Low |
| Total muscle mass in kg | Eg | MD | 1 | No serious risk of bias_5_ | Not serious_7_ | Not serious | Very serious_7,_ _12,_ _20_ | Not serious_24_ | **IG** 54 | **CG** 50 | -0.8 (-1.32 to 0.28) | 0.003 | n.a.* | Low |
| **WB-EMS + Protein Supplementation** | | | | | | | | | | | | | | |
| Body fat in % | H-C | MD | 2 | No serious risk of bias_1_ | Serious_9,_ _e_ | Not serious | Serious _12,_ _18_ | Not serious_23_ | **IG** 58 | **CG** 59 | -1.27 (-3.33 to 0.79)_★_ | 0.23 | 94% | Low |
|  | Yin | MD | 2 | No serious risk of bias_21_ | Very serious_28,_ _e_ | Not serious | Serious_12,_ _18_ | Not serious_25_ | **IG** 58 | **CG** 59 | -1.27 (-3.33 to 0.79) | 0.23 | 94% | Very low |
| Total body fat mass in kg | H-C | MD | 1 | No serious risk of bias_1_ | Not serious_7_ | Not serious | Very Serious_7,_ _12,_ _14,_ _20_ | Not serious_23_ | **IG** 33 | **CG** 34 | -2.01 (-2.82 to -1.20)_★_ | <0.0001 | n.a.* | Low |
| ASMM in kg | H-C | MD | 1 | No serious risk of bias_1_ | Not serious_7_ | Not serious | Very serious_7,_ _12,_ _19_ | Not serious_23_ | **IG** 33 | **CG** 34 | 0.44 (0.20 to 0.68)_★_ | 0.0003 | n.a.* | Low |
|  | Yin | MD | 1 | No serious risk of bias_21_ | Not serious_7_ | Not serious | Very serious_7,_ _12,_ _14,_ _19_ | Not serious_25_ | **IG** 33 | **CG** 34 | 0.46 (0.22 to 0.70) | 0.0002 | n.a.* | Low |
| SMMI | H-C | SMD | 2 | No serious risk of bias_1_ | Not serious_9,_ _11_ | Not serious | Serious_12,_ _19_ | Not serious_23_ | **IG** 58 | **CG** 59 | 0.72 (0.22 to 1.23)_★_ | 0.005 | 43% | Moderate |
|  | Yin | SMD | 2 | No serious risk of bias_21_ | Not serious_8_ | Not serious | Serious_12,_ _14,_ _19_ | Not serious_25_ | **IG** 58 | **CG** 59 | 1.18 (0.78 to 1.57) | <0.0001 | 0% | Moderate |
| Grip strength | H-C | MD | 2 | No serious risk of bias_1_ | Not serious_8_ | Not serious | Serious_12,_ _18_ | Not serious_23_ | **IG** 58 | **CG** 59 | 1.10 (0.30 to 1.90)_★_ | 0.007 | 0% | Moderate |
|  | Yin | MD | 2 | No serious risk of bias_21_ | Not serious_8_ | Not serious | Serious_12,_ _19_ | Not serious_25_ | **IG** 58 | **CG** 59 | 1.31 (0.50 to 2.11) | 0.001 | 0% | Moderate |
| Gait speed in m/s | H-C | MD | 2 | No serious risk of bias_1_ | Not serious_8_ | Not serious | Serious_12,_ _18_ | Not serious_23_ | **IG** 58 | **CG** 59 | 0.04 (0.02 to 0.06)_★_ | 0.0001 | 0% | Moderate |
|  | Yin | MD | 2 | No serious risk of bias_21_ | Not serious_8_ | Not serious | Serious_12,_ _19_ | Not serious_25_ | **IG** 58 | **CG** 59 | 0.04 (0.02 to 0.06) | 0.0001 | 0% | Moderate |

Abbreviations: IG = Intervention Group, CG = Control Group, CI = Confidence Interval, A= Author; Eg = Eglseer et al. 2022, H-C= Hita-Contreras et al.2018, Hs= Hsu et al. 2019, Yin=Yin et al. 2020; MD = Mean Difference; SMD = Standard Mean Difference

* n.a. = not applicable

_!_ IG was corrected from *n* = 6 to *n* = 36 according to the primary study by Kim et al.2016, and the effect was newly calculated using the mean and SD according to Hita-Contreras et al. 2018

_★_ Effect size was calculated using RevMan 5.4.

**_1_** 2 out of 7 studies have high risk of bias in terms of participant blinding, but blinding is difficult in resistance training, and the outcome was objectively measured, and the overall risk of bias was felt to be very low. Therefore, we did not downgrade.

_2_ No study had an allocation concealment, and 3 of 14 studies had inadequate follow-up. Therefore, we downgraded it (PEDro).

_3_ More than 50% of the included studies had no concealed allocation, participant blinding, therapist blinding, and/or intention to treat. Therefore, we downgraded it (PEDro).

_4_ Mean PEDro score was 5.6, and no detailed information on the different types of bias are available. Therefore, we decided to downgrade it (PEDro).

_5_ Only 1 out of 12 studies had high risk of attrition bias, and the overall risk of bias was felt to be very low. Therefore, we did not downgrade.

_6_ I_2_ is 90%. Therefore, we downgraded it

_7_ Only one study.

_8_ CI overlapping, *I*_2_ 0%.

_9_ CIs overlapping.

_10_ CIs partially overlapping.

_11_ Heterogeneity (*I²* > 0 ≤ 50%).

_12_ Small sample.

_13_ No significant primary study.

_14_ Narrow CI.

_15_ No overall effect.

_16_ Wide CI.

_17_ Very low sample size.

_18_ 50% of studies had a significant effect.

_19_ Overall significant effect.

_20_ Overall effect not significant.

_21_ Only 2 out of 12 studies had no blinding of participants and personnel, 1 study had no allocation concealment and incomplete outcome data.

_22_ Publication bias was not mentioned, no Funnel Plot or Egger’s test conducted, no search in study registers.

_23_ Egger’s test p > 0.05 for all outcomes.

_24_ Number of studies was too low to perform Funnel Plots, but no indication that publication bias was detected.

_25_ No Funnel Plots, but publication bias for individual studies was low; search conducted in trial registries and 6 databases.

_26_ 1/3 of primary studies showed a significant effect.

_27_ Wide variance of point estimates across studies.

_28_ No overlap of CI.

_29_ Included studies show opposite effects.

_30_ Nearly significant effects reported in each primary study.

_31_ Wide CI in 2/3 of primary studies.

_32_ Primary studies in favour of treatment.

_33_ 2/3 primary studies significant.

_34_ ¼ of primary studies showed no effect, 1/2 studies had a broad CI.

_35_ 2/5 primary studies significant.

_36_ 3/5 primary studies significant.

_37_ One primary study with wide CI.

_a_Heterogeneity (*I²* > 50 < 75%), _b_high risk for detection bias, _c_CI overlaps with no-effect line, _d_high risk for detection and attrition bias, and _e_considerable heterogeneity (*I²* > 75%).
